# Pennsylvania Hospital

**Published:** 1838-09-12

**Authors:** J. Forsyth Meigs

**Affiliations:** Resident Surgeon


### CLINICAL REPORTS.

### PENNSYLVANIA HOSPITAL.

[Reported by J. Forsyth Meigs, M. D., Resident Surgeon.]

*Case of Dislocation of the Femur upon the Dorsum Ilii, occurring from a fall in a child, eleven years of age.*

Admitted August 26th, 1838, J. B., æt. eleven years, with a dislocation of the right femur upon the dorsum ilii, caused by a fall from a cart eighteen hours previously. When brought in, he complained of excessive pain and soreness about the hip, and would scarcely allow it to be touched. On placing him in the erect posture, it was found that he rested his weight entirely upon the left limb, the other being slightly flexed upon the pelvis, the foot turned inwards, with the ball of the great toe resting upon the tarsus of the sound foot. The thigh could be flexed upon the pelvis but not extended; it could be rotated inwards, but not outwards; and could be adducted, but not abducted. There was a considerable prominence on the right buttock; the trochanter was drawn upwards, and by rotating the limb, the head of the bone could be distinctly felt upon the dorsum. On measuring from the anterior superior spinous process of the ilium to the internal malleolus, the injured limb was found to be an inch and a half shorter than the other. Some tartar emetic being administered, the luxation was readily reduced by means of a counter-extending band placed in the perineum, while extension was made by three men from just above the knee, the bone slipping into its place with a snap in the space of two minutes; when the protuberance about the hip disappeared, the thigh could be extended, and by measurement was found to be of exactly the same length as the other. The limbs were secured by means of a roller, and placed at rest; the hip was afterwards leeched, and cold applied. In six days the boy was able to walk, and has since been discharged well.

This case is interesting from the fact that dislocations are of rare occurrence in children, excepting as a consequence of previous disease of the joints. Sir A. Cooper says, "young persons are also (speaking previously of old) very rarely the subjects of dislocations from violence; but now and then such cases do occur." He mentions an instance of dislocation in a child of seven years, and one in a boy of thirteen years, which were both those of the hip upon the dorsum ilii, in the latter being complicated with a fracture of the cervix femoris. Liston mentions no cases, neither does Boyer or John Bell.

---

*List of Accidents, admitted into the Pennsylvania Hospital, from August 22d, to September 5th,* 1838.

A case of lacerated wound of the hand, in a boy fourteen years old, from the bursting of a gun; thumb completely separated from the trapezius, and adhering to hand merely by a few tendons; palm much lacerated, the flexor tendons laid bare with a wound three inches in extent on front of fore-arm. When admitted there was considerable hæmorrhage; some small vessels were secured, the radial artery was cut upon and tied; wound brought together by adhesive strips; lint applied, arm placed on a splint, and kept moist by cold lead water by means of a syphon; since had some fever for which took an effervescing draught and a purge; at present dressed with bran, suppurating and doing well; good diet.—A case of fracture of the fibula of the right leg, about two inches above the ankle, from a fall from a height; some deformity, foot being turned outwards; fracture reduced, and leg placed in a fracture-box which retains the ends in apposition; lead-water applied; doing well.—A case of severe contusion of both ankles, with a slight wound in one, from being jammed in between two rail-road cars; when admitted, there was extensive swelling from effusion into the joints, and great pain; legs placed at rest and cold lotions used; doing well.—A case of punctured wound, from the falling of an iron rake, one of the teeth of which entered the hand at the junction between the first metacarpal bone and the wrist, joint not opened; when admitted, parts swollen and tender, with much pain through whole hand; placed on a splint, and cold applied; since discharged cured.—A case of fracture of the clavicle within the sternal third, from a fall upon the shoulder; dressed with hospital apparatus, since attacked with symptoms of mania-a-potu, for which he had appropriate treatment; now doing well.—A case of injury to the brain, from a blow; when admitted was insensible, with pupil of one eye widely dilated, other not so much so; cold to head, sinapisms to extremities; stimulating enema; afterwards cupped, blister to back of neck; died in four days.—A case of fractured femur in a boy fourteen years old, from attempting suddenly to escape from one of his companions, in doing which he fell and broke his thigh; dressed with Dr. Physick's modification of Desault's apparatus; since had considerable effusion and swelling; now doing well.—A case of fracture of the body and spinous process of one of the lower dorsal vertebræ, with fracture of the lower ribs of both sides; no paralysis ensued; extensive emphysema from a wound of the left lung by the rib; no pneumothorax, owing to previous adhesions from pleurisy; there was also rupture of the liver and spleen, the man dying in five days from peritonitis caused by the effusion of blood in the cavity of peritoneum.—A case of compound fracture of the great toe of the left foot from the falling of a heavy stone on it; there was an extensive wound with the upper fragment projecting; hæmorrhage not very great, arrested by the application of lint, and slight pressure; leg placed at rest, and at present cold lead-water made use of by means of a syphon. A case of contusion of the scalp from the falling of a brick; there was considerable swelling from effusion of blood; no fracture; patient a man of rather bad habits; placed in bed; cooling lotion applied to head; since attacked with symptoms of inflammation of brain for which cupped and purged; now doing well.—A case of lacerated wound of the scalp, caused by a fall; when admitted, the man was bleeding profusely; a wound about half an inch in extent was found upon superior portion of the occipital bone, where there existed an extensive thrombus, which was emptied by pressure, and the hæmorrhage arrested by a dossil of lint and pressure; since removed by friends.—A case of fracture of the leg in a boy eleven years old, caused by a fall from a height; dressed in usual manner.—A case of partial fracture of the right leg in a boy eleven years old, who has three times before been in the house for the same accident; the leg is much bent inwards, owing to his having been removed too soon by his friends in one of the previous cases, and allowed to use his limb before it had become firm; at present there exists a good deal of inflammation and tenderness, for which cold is used, and rest enjoined.—The case of lacerated hand and fingers mentioned in last report still remains in the house; the sloughs have separated, leaving more than half the surface on the front and back of the fore-arm entirely divested of integument, the tendons of the thumb laid bare, and the wrist-joint opened, with profuse discharge of pus; at present dressed with bran; under full diet with quinine and porter.—The case of compound fracture of toes is doing well; bone becoming firm, external wound nearly united, dressed with strips and cerate.

